# Attitudes of Black American Christian church leaders toward Opioid Use Disorder, overdoses, and harm reduction: a qualitative study

**DOI:** 10.3389/fpsyt.2024.1359826

**Published:** 2024-04-03

**Authors:** Akosua B. Dankwah, Richard B. Siegrist, Ira B. Wilson, Michelle McKenzie, Josiah D. Rich

**Affiliations:** ^1^ Department of Psychiatry, Recovery Research Institute, Massachusetts General Hospital and Harvard Medical School, Boston, MA, United States; ^2^ Department of Health Policy and Management, Harvard T.H. Chan School of Public Health, Boston, MA, United States; ^3^ Department of Health Services, Policy and Practice, Brown University School of Public Health, Providence, RI, United States; ^4^ Department of Medicine, Warren Alpert Medical School of Brown University, Providence, RI, United States; ^5^ Center for Biomedical Research Excellence (COBRE) on Opioids and Overdose, Rhode Island Hospital, Providence, RI, United States

**Keywords:** Opioid Use Disorder, opioid overdose, Black American, qualitative, Christian, church leaders, harm reduction, clergy

## Abstract

**Introduction:**

Black American Christian church leaders are trusted community members and can be invaluable leaders and planners, listeners, and counselors for Opioid Use Disorder (OUD) sufferers in the opioid overdose crisis disproportionately affecting the Black community. This qualitative study examines the extent to which the knowledge, attitudes, practices, and beliefs of Black American church leaders support medical and harm reduction interventions for people with OUD.

**Methods:**

A semi-structured interview guide was used to conduct in-depth interviews of 30 Black Rhode Island church leaders recruited by convenience and snowball sampling.

**Results:**

Thematic analysis of the interviews identified four themes: Church leaders are empathetic and knowledgeable, believe that hopelessness and inequity are OUD risk factors, are committed to helping people flourish beyond staying alive, and welcome collaborations between church and state.

**Conclusion:**

Black American Christian church leaders are a critical resource in providing innovative and culturally sensitive strategies in the opioid overdose crisis affecting the Black American communities. As such, their views should be carefully considered in OUD policies, collaborations, and interventions in the Black American community.

## Introduction

The United States is experiencing a drug overdose crisis (1). The February 2022 Standford-Lancet Commission on opioid use in North America reported that opioid overdoses had caused more than 600,000 deaths in the United States and Canada since 1999 (2). By the end of the decade, the number of recorded deaths is projected to double (3). Near the mid 2010s, the opioid overdose crisis shifted from occurring predominantly among Whites to being essentially a crisis among Black American communities (4) due mainly to fentanyl-involved overdose deaths (5). Furthermore, over the years, while there has been a medicalization of drugs among White American communities, there has been widespread and disproportionate criminalization of drugs among Black Americans, with Black Americans 6-10 times more likely to be incarcerated for drug offenses compared to White Americans (6).

In Rhode Island, opioid overdose deaths for non-Hispanic Blacks rose from 26.4 to 49.3 per 100,000 from 2019 to 2020, the highest of any racial/ethnic group in the state (7). Rhode Island’s implementation of the first statewide program in 2016 to provide Medications for Opioid Use Disorder (MOUD) to incarcerated persons (8) with associated marked reductions in post-incarceration opioid overdose fatalities (9), was insufficient to reduce the 2019 to 2020 high opioid overdose deaths. The fentanyl contamination of the non-opioid drug supply was a contributing factor. The rate of fentanyl-involved fatal overdoses was highest among non-Hispanic Black Rhode Islanders compared to Hispanics and non-Hispanic Whites, with rates of 47.8, 20.4, and 24.8 per 100,000, respectively, in this period (7). Thus, almost all non-Hispanic Black deaths in the state associated with opioids involve fentanyl.

In response to fatal overdoses in the state, the Rhode Island government established the Governor’s Overdose and Prevention Taskforce in 2015 (10), comprising experts and community members. The Taskforce brings together professionals and community members working together to prevent overdoses and save lives (11). Additionally, the Rhode Island legislature passed, and the Governor signed a law permitting the first state-sanctioned pilot harm reduction centers in the country in July 2021 (12). To engage Black Americans and other minority populations, the Rhode Island government has adopted Connecticut’s Imani Breakthrough Recovery Intervention (13), a culturally informed approach to engaging Blacks and Latinos in substance use treatment based in churches of color.

The Black American church has been vital in disseminating other public health interventions (14), such as HIV prevention and education (15), influenza immunizations (16), and COVID-19 vaccinations (17). In addition to hosting 12-step groups like Alcoholics Anonymous (18), churches have promoted recovery from addiction efforts with Christian-based support groups, including Adult and Teen Challenge USA (a residential recovery program) and Celebrate Recovery (an evangelical Christian 12-step recovery support program) (19). While faith-based organizations could partner in expanding harm reduction services for high-risk populations, little is known about their attitudes and practices regarding OUD.

The first author interviewed Black Rhode Island Christian clergy to address the research question: What is the extent to which the knowledge, attitudes, practices, and beliefs of Black American church leaders support medical and harm reduction interventions for people with opioid use disorder (OUD)? The authors hope this study will inform and encourage church-based opioid overdose interventions, such as the Imani Breakthrough Recovery Intervention project underway in Rhode Island.

## Methods

### Research design overview

This qualitative research study used a semi-structured interview guide to conduct 30 interviews of Black Rhode Island church leaders recruited by convenience and snowball sampling.

### Participants

**Table 1** summarizes the demographic information and other characteristics of the church leaders. There were 19 (63%) male and 11 (37%) female church leader participants. The majority (63%) of participants were between 50 and 65 years. Half of them had no more than a college degree, and the other half had graduate degrees. Many were bi-vocational and had jobs besides their pastoral ministry, including a property manager, a community health worker, a chemist, clinical providers, and those serving in government agencies. Most congregants resided in Providence County of Rhode Island. Others lived in Massachusetts and Connecticut. Most congregations were Pentecostal. Others were Interdenominational, Church of God, African Methodist Episcopal, Nondenominational, American Baptist, Assemblies of God, and Evangelical. Most churches were multiracial, with a Black American predominance. Their congregant ethnicities included Caribbean, Cape Verdean, Liberian, Ghanaian, White American, and Native American. Additionally, congregation sizes ranged from nine to about 500. However, most congregations had less than 100 members.

**Table 1:** Characteristics of Black Rhode Island clergy interviewed about their attitude toward Opioid Use Disorder and harm reduction October 2021-January 2022.

Characteristic	Participants, No. (%) (N = 30)
Age, years	
<50	5 (17)
50-65	19 (63)
>65	6 (20)
Sex	
Women	11 (37)
Men	19 (63)
Education	
College/Some college	15 (50)
Masters	8 (27)
Doctorate	7 (23)
Christian Denomination	
Pentecostal/Charismatic	13 (43)
African Methodist Episcopal	5 (17)
Inter/Nondenominational	6 (20)
Other	6 (20)
Racial/ethnic identities	
African American	11 (37)
African American and other ethnic identities*	19 (63)

SOURCE Authors’ analysis of study data. NOTE Other ethnic identities include “black,” Jamaica, Nigeria, Ghana, Zaire, American Indian, “Afro Trinidadian,” Cherokee Blackfoot Native American.

### Procedures

The church leaders were recruited by convenience and snowball sampling methods. For instance, at an event introducing the Imani Breakthrough Intervention to the Rhode Island public, the first author was introduced to the Ministers Alliance of Rhode Island, a multicultural collaboration of Christian pastors and ministers in the state. Soon afterward, the first author delivered a presentation about the study to the Alliance at their monthly meeting and obtained 15 participants following the presentation. Many of these participants connected the first author to their colleagues to expand the pool of participants. The first author followed up with subsequent participants via emails and phone calls.

The inclusion criteria were Black clergy in predominantly Black churches, ages 18+ years, who were pastors or in other leadership positions in Rhode Island churches. The Harvard Longwood Campus Research Protocol Institutional Review Board granted exempt status for the research protocol.

Since completing the project, the first author has outreached to some clergy participants during several monthly meetings at the Alliance for their feedback on the study outcomes, and they supported the findings.

### Data collection

An introductory email to participants included a request for their availability to participate in the research interview and an attachment of the consent document with the project details. A positive email response and verbal consent before the interview sufficed because of the study’s exempt status. The authors developed a semi-structured interview guide (**Supplementary 1**) about the OUD crisis and harm reduction by drawing from previous related studies and content area experts. The first author ran the interview guide past a qualitative research and content area expert and updated it per their feedback before administering it. The interviews were conducted via the Zoom platform and included questions for the participants on the following topics:

- Understanding the role of the interviewee within the organization and gathering details about the organization itself.
- Knowledge of the OUD crisis and beliefs
  iii. Knowledge of harm reduction techniques and beliefs about their efficacy and appropriateness
  iv. Opinions and experience with faith-based intervention methods to mitigate the opioid use disorder crisis.
  v. Demographic information of the church leaders and congregations

### Data analysis

The first author followed a thematic analytic approach (20). 3Play Media (21) transcription service was employed to generate interview transcripts to aid in data familiarization and noting initial impressions. The data was managed with Dedoose qualitative analysis software (22) using an iterative analytic process with deductive codes capturing topics from the semi-structured interview guide and inductive codes derived from concepts relevant to the study, such as culturally informed approaches from the Imani Breakthrough Recovery Intervention literature. Codes and subcodes were refined iteratively by merging similar codes and associated quotes and regrouping codes and subcodes accordingly. The resulting codes were collated to identify underlying concepts and impressions to generate statements articulating themes. Further, compelling quotes were selected (see Results section) to highlight the themes.

### Transparency and openness

The authors follow the journal article reporting standards for qualitative research design (23). Given how small the number of Black American clergy are in Rhode Island, study data, including recordings and interview transcripts, cannot be shared. Moreover, due to the deductive disclosure (24), it is almost impossible to de-identify the recordings and transcripts generated. As such, data sharing would breach the confidentiality implied in the participation agreement. To make this study transparent, however, the authors have included the semi-structured interview guide (**Supplementary 1**), resulting codes and subcodes generated (**Supplementary 2**), and thematic codes and subcodes with selected responses and associated Biblical references (**Supplementary 3**) as **Supplementary Information**. This study’s design and its analysis were not pre-registered.

To minimize bias, the semi-structured interview guide questions (**Supplementary 1**) were phrased in a neutral way to invite a conversation rather than yes-no responses. In addition, the guide used accessible language with terms defined as needed.

## Results

30 church leaders were interviewed from October 18, 2021, to January 4, 2022. The Zoom interviews lasted 70 minutes on average. All church leaders referenced Biblical texts. **Supplementary 3** summarizes codes generated from the thematic analysis with selected responses and associated Biblical references (25).

### Themes

The following four themes and selected related responses were identified from the analysis:

*1. Church leaders are empathetic and knowledgeable about the OUD crisis in their communities.* Church leaders’ knowledge, empathy, and compassion regarding the OUD crisis stem from their lived experiences. Some church leaders acknowledged the utility of Celebrate Recovery and Adult and Teen Challenge USA interventions for persons with substance use disorders. Other church leaders were involved in local church recovery support ministries and other ministries supporting social determinants of health by providing housing, education, medical care, employment opportunities, food, and clothing to the church and community.

A church leader who suffered a near-death experience with OUD and who had lost a sibling from an opioid overdose recounted how her faith contributed to her survival and subsequent recovery. Now, the majority of her congregants are persons grappling with substance use problems. As she recounted,

“I am constantly trying to remind people He (Jesus) did it for me. He can do it for you. I could not have done anything myself.”

Similarly, another church leader shared her experience of losing family members from drug overdose:

“It has even affected my own family. I’ve had three uncles die from drug overdoses.”

While a few church leaders with clinical backgrounds (including three clinical providers) were medically knowledgeable about OUD, other church leaders had contextual and historical OUD knowledge regarding the opioid overdose crisis in their communities. For many church leaders, the opioid crisis in their communities was not new:

“For the Black community, including the Black church, this is nothing new. We have been dealing with it for decades and decades and decades. Because we’re not talking about people who are out there. We’re talking about our sons and daughters. We’re talking about our fathers and mothers. We’re talking about our cousins. We’re talking about Sister So-and-so, who lives a couple of doors down.”

The church leaders understood culturally competent care. They know their community and understand what they need and are not getting. A leader’s narrative demonstrates the significance of engaging experts who can better relate to their community needs in providing OUD intervention:

“Some of these clinicians can’t relate to that. So they don’t understand. So we need those programs to help our people. But we need our people, we need the people that come from here, who knows this guy’s mother, knows this guy’s grandmother, to convince them to get involved, and help them, and wrap their arms around them, and bring it back to the way it used to be. And where do we find a lot of those people? In our churches.”

*2. Church leaders believe that hopelessness and inequity are OUD risk factors*. All clergy alluded to hopelessness as OUD drivers, including the trauma individuals and families experienced from the COVID-19 pandemic:

“I always believe that it’s a multifaceted issue. And with impact of the pandemic, particularly unemployment, additional stress, financial strain on families, and sense of hopelessness, it’s almost like what some might describe as the perfect storm for pushing certain people who may not have many healthier support options.”

The interviews also had many undertones of the inequity and injustice experienced in the Black American community when accessing OUD treatment facilities. For example, a church leader’s expressions suggested that some of their community members accessing OUD treatment facilities were ill-treated and not administered care with compassion, indicative of the concept of criminalization rather than the concept of medicalization:

“Black communities’ reception at these clinics and hospitals is among the poorest. When a Black person with addiction goes to the clinic for help, sometimes that is where they tend to see themselves as drug abusers, but not as people who have health problems.”

Moreover, these perceptions of poor reception when accessing OUD treatment facilities, coupled with inequity experienced by the Black community, further discourage them from accessing resources for OUD:

“First of all, as Black people, we do not really believe in a system that was not designed for us. And when you’re already disenfranchised, and you’re already down, you’re already marginalized, it’s not like you want to go and be mistreated again.”

*3. Church leaders are committed to helping people flourish beyond staying alive.* Generally, the church leaders appreciated the benefits of MOUDs and harm reduction methods, such as naloxone, that would set OUD individuals on a path to remission and thriving, not only keep people alive. All advocated for counseling and preventive harm reduction methods, including MOUDs. For instance, a church leader’s response to his opinion about faith-based intervention methods to mitigate the OUD crisis was:

“I will use faith to believe in God and bring professional counselors and medical experts to help folks using these drugs. I believe in the treatments, and I believe that the same God who changes and touches lives also gives us knowledge and wisdom to treat people so that people will get out of drugs. Because the thing has both physical and spiritual aspects, we need to also deal with the spiritual elements: prayer, counseling, and believing God to touch them.”

However, most of the church leaders were conflicted over those harm reduction interventions that enabled the confident utilization of opioids, such as clean syringe exchanges and fentanyl test strips and the establishment of harm reduction centers. Their teachings and convictions did not favor misusing opioids or any other substance that would jeopardize a person’s health and well-being. A church leader was almost to the point of tears sharing about her conviction:

“Would you encourage your child to use the test strip first, or are you trying to bring them to Christ? We’re supposed to bring people to do what’s godly. We can’t do that while inventing new ways to cheat.”

They were also convinced that the church establishment is critical in addressing the spiritual and deep-rooted issues of meaning, purpose, and value, allowing people to live and flourish beyond MOUDs and harm reduction methods. For instance, a church leader shared about his conviction:

“The church has a responsibility. I believe in addressing it from a holistic perspective. We are the only entity on the planet with the right and authority to deal with the whole person, the spirit, the soul, and the body, right? Because in my understanding, in my position as a spiritual leader, not every problem is spiritual. What is spiritual, you address it spiritually. What is medical, address it medically. Now, if it is a combination of both, you use both to address it.”

Further, beyond MOUDs and harm reduction methods that keep people alive, church leaders would prioritize prevention efforts such as assisting individuals with their social determinants of health needs such as safe housing and communities, better living conditions, and access to resources to enable individuals and families to flourish. A church leader with personal lived experience on the streets, now ministering to homeless persons, many of whom suffer from OUD, shared:

“I think the homeless crisis is a major start to getting some of these people out of the streets into a safe environment where they can pick up the pieces in their lives. I don’t mean put them in the projects that are already infested with drugs, somewhere where they can feel good about living, and look around, and say, OK, now I have somewhere to store my medication. I have somewhere where I can get mail, you know? I have somewhere to put on clean clothes, get up, and go to a job interview. You’re not going to get that in a shelter, you know?”

*4. Church leaders welcome collaborations between church and state.* While all the church leaders were pleased with the Rhode Island government’s endorsement of the Imani Recovery Breakthrough Intervention, some expressed a disconnect while engaging with the Rhode Island Governor’s Overdose and Prevention Taskforce. A church leader who was invited to a Taskforce meeting felt out of place:

“I remember the first time I went there [Governor’s Overdose and Prevention Taskforce]; I felt like I was out of the league. I wasn’t in. You know what I mean? I think it was more of the medical field, with the social workers, and with others. So I sat there for a few minutes, and I’m going, you know what, this is not what I should be part of. And I tried again, and it was still the same. So I think they would be more involved if they made faith leaders comfortable.”

Nevertheless, the church leaders are open to partnering with state authorities around the OUD crisis. They are also open to collaborating with state authorities around the OUD crisis. According to a church leader:

“Spiritual care providers or clergy and lay leaders are not in competition with professional health care providers. Our work is complementary to the work of professional healthcare providers. Fighting opioid addiction requires an intentional, integrated effort by both spiritual and secular community leaders.”

Church leaders will fully engage with state authorities and leaders in OUD intervention efforts when they are given autonomy and their values are recognized and appreciated, as elaborated by this church leader:

“Empowering the Church with additional tools and resources, including training and specific education, is a step in the right direction. The false objection based upon the concept of the separation of church and state must not be brought into the conversation. If so, the Church-Faith-Based approach will be anemic and impotent. Faith-Based and Church-Faith-Based frameworks should be seen as different approaches. The former includes the discussion of God, while the latter could involve the Head of the Church - Jesus Christ, His Word [the Biblical scriptures] and the Holy Spirit.”

## Discussion

This qualitative study of 30 semi-structured interviews of Black Rhode Island church leaders examined their views toward Opioid Use Disorder and harm reduction. The major finding of the study was that Black American church leaders deeply care about the health and wellbeing of people with Opioid Use Disorder. While the Black American church leaders are concerned about interventions they perceive as perpetuating use, such as fentanyl test strips or sterile syringe exchanges, they are open to many harm reduction interventions, including counseling, Medications for Opioid Use Disorder, and naloxone. The themes emerging from the interviews highlight opportunities to engage church leaders who are highly respected and influential community members to address the ongoing opioid crisis in Black communities.

The theme, *Church leaders are empathetic and knowledgeable about the OUD crisis in their communities*, resonates with Jerome Adams, MD, MPH, previous US Surgeon General, who appreciated the critical role of the faith community in approaching the Opioid Crisis in their communities with compassion and a sense of a call to duty. In his statement, “Keeping Faith, Bringing Hope and Healing in the Midst of the Opioid Crisis,” the Surgeon General recognized the contributions of faith communities along with social service agencies in encouraging access to MOUDs while promoting recovery services and prevention of substance misuse (26). The personal, familial, and medical close encounters with opioid overdoses by several church leaders developed empathy toward persons with OUD and provided contextual knowledge about the OUD crisis. Moreover, some church leaders in this study were involved in substance use prevention and recovery efforts locally in their congregation and through widespread Christian-based recovery ministries such as Adult and Teen Challenge USA, a residential recovery program. Adult and Teen Challenge USA employs a Bible-based curriculum to aid individuals in their recovery journey holistically (psychologically, socially, physically, and spiritually) (27). The economic benefit of faith-based residential recovery programs is significant. An impact evaluation study to assess the financial impact of a faith-based long-term residential addiction recovery intervention, the Mission, in Baltimore, Maryland, revealed that every person who participates in the Mission for a year saves the state and county governments $14, 263. These savings result from less spending on health care, social services, and criminal justice utilization (28). The study participants comprised 5,122 homeless men recovering from substance use disorder from 2006 to 2019. Surmising from this impact evaluation study, Adult and Teen Challenge USA, would be a cost-effective recovery intervention.

In addition to their lived experiences, several church leaders provided historical accounts of the OUD crisis, emphasizing that the OUD crisis was not a new epidemic in the Black American community. Historically, Black Americans accounted for the majority of the heroin use disorder crisis of the 1960s and 1970s when drug laws against this heroin-related opioid epidemic predominantly affected Black American and minority communities, resulting in a disproportionate prison population legacy (29, 30). Relatedly, the profile of heroin users in the 1960s and 1970s were minority males (31). The disparate incarceration of minority populations persists in more recent times. For example, in 2001, 94% of imprisoned drug offenders in New York were Blacks and Hispanics compared to 5.3% Whites (32). The criminalization of minority heroin opioid users is in stark contradiction to the medicalization approach to OUD, viewing the opioid crisis as a public health problem when it is also plaguing Whites (33).

Moreover, financial restraints leading to inadequate health insurance, distrust of the medical community, and healthcare infrastructure from historical mistreatment and stigma are barriers to assessing healthcare services for OUD (29). These barriers lead to the second theme: *Church leaders believe that hopelessness and inequity are OUD risk factors.* Furthermore, consistent with the sentiments expressed by some church leaders, research has revealed the ethnic-discordant relationship between Blacks and health professionals, leading to Blacks disliking their experiences with their medical care compared to Whites (34).

Unfortunately, despite the shift in the focus of the epidemic from criminalizing it to treating it, the black population is still impacted disproportionately compared to their white counterparts (29, 35). Researchers have attributed the disparity in OUD treatment outcomes for black populations to omitting them from discussions around the OUD epidemic (29, 35) an indication that providing OUD treatments and intervention strategies to Black and other minority populations should take cultural competence into account.

All the church leaders advocated for primary prevention and recovery strategies such as counseling and role modeling to safeguard youth and families from engaging in substance misuse. They also advocated for supporting people in recovery, especially in addressing trauma and life challenges. The clergy would rather commit to providing these support strategies to enable people to flourish beyond merely keeping them alive through medical interventions. The study participants’ prevention and recovery outlook for OUD was consistent with researchers (36), who demonstrated that spiritual assistance and religious participation can help prevent misuse of substances in young adults and aid persons in addiction on their recovery journey. Their findings mirrored other studies demonstrating associations between spiritual practices and recovery from substance use disorder (37) and showing relationships between perceived spiritual support and increased self-efficacy and less cravings from substance use disorder (38). These outcomes are associated with hope. Specifically, a study investigated the association between distress tolerance and general and religious or spiritual hope for ethnic minorities, including Blacks, compared to non-Hispanic Whites, in a nationally representative adult sample (N=2875) (39). The researchers showed that ethnic minorities generally experienced lower degrees of psychological distress compared to non-Hispanic whites. Moreover, the ethnic minority groups, including Blacks, experiencing lesser degrees of psychological distress indicated higher degrees of religious or spiritual and secular hope compared to non-Hispanic Whites who are intolerant to distress.

The religious or spiritual orientation of hope by ethnic minority groups is critical in coping with challenges beyond their capacity to contain (40), including the inequities and injustices that Blacks face compared to Whites in the face of the OUD crisis. Against this backdrop is the third theme: *Church leaders are committed to helping people flourish beyond staying alive.* For the clergy, providing OUD treatment options and interventions alone is inadequate if they do not offer holistic care for the people. They believed that their faith and religious practices could address the deep-seated needs of people suffering from substance use disorders (19), including OUD.

Stemming from this viewpoint, most of the clergy discouraged the use of harm reduction methods they perceived as perpetuating use, including clean syringes and fentanyl test strips. This finding was congruent with an online survey of (n=133) faith leaders’ views of a needle exchange program in Illinois (41). Per the survey, the faith leaders supposed that the needle exchange program would increase drug use, though they also appreciated that the needle exchange programs would reduce blood-borne infections. This mixed response to the study findings is also captured in the present study as the “Inner Value Conflict” subcode. (Please see **Supplementary 3** for more selected responses and associated Biblical references).

The overwhelming support for the church-based drug overdose intervention, the Imani Breakthrough Recovery Intervention, endorsed by the Rhode Island government, is suggestive of the fourth theme: *Church leaders welcome collaborations between church and state.* The Imani Breakthrough Recovery Intervention is a community-based participatory research (CBPR) approach to address the rising cases of drug overdose among Blacks and Latinos, began in partnership with the State of Connecticut Department of Mental Health and Addiction Services, funded by the Substance Abuse and Mental Health Services Administration (SAMHSA) (42). Parishioners and persons with lived experience facilitate the innovative intervention run by Black and Latino churches. The 22-week intervention is unique in addressing the effect of trauma and racism experienced by these racial minority communities relative to substance use disorder, including OUD. The intervention addresses the inequities in social determinants of health (SODH) encountered by these communities by providing wraparound support and life coaching while focusing on SAMHSA’s eight dimensions of wellness (physical, intellectual, environmental, spiritual, social, occupational, emotional, and financial health) (43) and the Citizenship Enhancement model brought about by determining the factors necessary for community reintegration by persons previously incarcerated or with mental illness. The Citizenship Enhancement model appreciates the need for access to employment opportunities, healthcare, housing, and a sense of belonging (44). A report showing 42% retention of Imani intervention participants at 12 weeks and data leading to a significant increase in Citizenship Enhancement scores from baseline to week 12, the Imani Breakthrough intervention shows promise in addressing the SODH disparities of people who use substances such as opioids in Black and Latino communities (42). The Imani Breakthrough is, thus, at the heart of the church leaders’ desire to enhance and rebuild lives to flourish and shows promise in providing culturally informed recovery intervention for Blacks and Hispanics.

These collaborations between faith-based organizations and state agencies are necessary to engage persons in addiction, their family networks, and communities in their recovery journey (45), who would otherwise be logistically unreachable by federal and state organizations. Furthermore, researchers have challenged the strict application of separation of church and state, allowing churches to use government funding to advance a social service without promoting religious activities (19). The researchers posit that this delineation between religious practices and government-funded social programs can lower the impact of faith-based interventions when the religious practices and beliefs are segregated from the intervention. Likewise, the Black church leaders in this study asserted the lower effect of faith-based initiatives not fully embracing their core beliefs.

This study adds to a growing body of knowledge showing the critical role of church leaders in addressing the OUD crisis in black and other ethnic minority communities. The church community is an intimate bridge connecting available interventions and resources to persons experiencing OUD, their families, and the community. Given that the success of OUD church-based initiatives is largely descriptive and anecdotal, more rigorous study designs, including independently cross-checking derived codes from different researchers and computing intercoder agreement, and mixed methods approach to triangulate data from different sources. Longitudinal and quantitative research methods, quantitative evaluation methods, cost-effectiveness, and economic impact assessments, should be employed to assess the public health utility of church-based interventions for OUD. Additionally, researchers should continue to include culturally centered, disparities reduction and community-engaged research approaches such as CBPR methods in interventions and study designs to empower Black and racially ethnic minority communities in discussions around the OUD crisis. These design methods can collectively provide innovative and targeted approaches for nontraditional partners to work together for high-risk groups in the fight against OUD.

Black church leaders have an affinity for primary prevention and flourishing in recovery strategies in OUD intervention efforts. Black church leaders are trusted community members and can be invaluable leaders, planners, listeners, and counselors for OUD sufferers. They are a critical resource in providing innovative and culturally sensitive strategies in the opioid overdose crisis affecting the Black American communities. Their views should be carefully considered in OUD policies, collaborations, and interventions in the Black American community.

This study’s limitations include convenience and snowball sampling recruitment methods that may be less representative of Black American clergy. While comparable to other qualitative research studies, it is worth noting that this sample was 30 clergy members and may not represent the perspectives of all Black clergy members. In addition, many of the churches were in Rhode Island’s Greater Providence community, which may have led to participants’ concerns about anonymity and confidentiality.

## Data availability statement

Given how small the number of Black American clergy are in Rhode Island, study data, including recordings and interview transcripts, cannot be shared. Moreover, due to the deductive disclosure, it is almost impossible to de-identify the recordings and transcripts generated. As such, data sharing would breach the confidentiality implied in the participation agreement. To make this study transparent, however, the authors have included the semi-structured interview guide (**Supplementary 1**), resulting codes and subcodes generated (**Supplementary 2**), and thematic codes and subcodes with selected responses and associated Biblical references (**Supplementary 3**) as **Supplementary Information**. Requests to access the datasets should be directed to AD, adankwah@mgh.harvard.edu.

## Ethics statement

The studies involving humans were approved by The Harvard Longwood Campus Research Protocol Institutional Review Board. The studies were conducted in accordance with the local legislation and institutional requirements. The ethics committee/institutional review board waived the requirement of written informed consent for participation from the participants or the participants’ legal guardians/next of kin because a positive email response to an informed consent document and obtaining verbal consent before the interview sufficed due to the study’s exempt status.

## Author contributions

AD: Conceptualization, Formal analysis, Methodology, Writing – original draft, Writing – review & editing. RS: Funding acquisition, Supervision, Writing – review & editing. IW: Supervision, Writing – review & editing. MM: Supervision, Writing – review & editing. JR: Conceptualization, Funding acquisition, Supervision, Writing – review & editing.
