# Supplementary material for: Attitudes of Black American Christian church leaders toward Opioid Use Disorder, overdoses, and harm reduction: a qualitative study
Source: Front Psychiatry. 2024 Apr 3;15:1359826. doi: 10.3389/fpsyt.2024.1359826 (PMC11021723; doi:10.3389/fpsyt.2024.1359826)
Supplement: Supplementary file 1 [file Table_1.pdf]

SUPPLEMENT 1

Semi-structured interview guide for Black Rhode Island clergy about their attitude toward Opioid Use Disorder and harm reduction.

A. PRE-INTERVIEW BRIEFING

1. Introductions and the purpose of the interview:

[Greetings and brief introductions]
To provide some study context, the rate of fatal overdose in Rhode Island has shot up to the highest level ever for the past two years. This rate has been disproportionately higher among our Black American communities, who now have nearly twice the rate of fatal overdose in Rhode Island compared to earlier rates. I am interested in exploring potential interventions with our Black communities. Over the next several weeks, I hope to interview Black American church leaders like you in Rhode Island to understand their attitudes and beliefs about the opioid crisis we are facing and if they can play a role in addressing it.

In addition, I am interested in any opinions you may have about the Imani Breakthrough Recovery Intervention, a church-based intervention to address the opioid crisis and other substance use problems that have been effective in Connecticut in the Black American and Hispanic communities. Rhode Island has initiated this intervention to mitigate the crisis in the Black American communities, and I am interested in how the state can successfully implement this intervention. I would also love to learn your views on harm reduction and medications for opioid use disorder.

2. Logistics and Verbal Consent

I appreciate your taking part today and want to let you know there are no right or wrong answers. I'd like to learn about your perspective and experiences.

Before we begin, could you review the consent form and let me know if it all makes sense? Do you have any questions?

- Are you okay with me recording our conversation?
    - Recording our conversation will enable me to analyze it and ensure I accurately capture all you've said.
- I'll be the only one listening to and transcribing this interview
    - I will remove any identifying details from our conversation (e.g., names of people, places, organizations)
- I will never share anything you say in connection with your name
- Are you okay with me using de-identified quotes from your interview in reports of this research?
- You are welcome to skip any questions you would rather not answer or stop the interview at any time. Please let me know if you don't understand any questions, and I will explain them to you.
- I want to reassure you that this interview is entirely confidential. My focus is on making sure that our communities are positively impacted as we learn more about the opioid overdose crisis.
- This interview is a private conversation, but I am sure you will be very interested in what other pastors say, and I am very committed to putting together a report that I can share with you.
- Is there anything else you want to discuss before we start?

SUPPLEMENT 1

Semi-structured interview guide for Black Rhode Island clergy about their attitude toward Opioid Use Disorder and harm reduction.

B. INTERVIEW GUIDE QUESTIONS

1. Rapport

- What is your congregation's name and location?
- What office/position do you serve?

2. Questions on knowledge regarding substance abuse and opioid overdose

[The rate of fatal overdose in Rhode Island has shot up to the highest level ever for the past two years. And this has been disproportionately higher among our Black communities, who now have nearly twice the rate of fatal overdose in Rhode Island compared to earlier rates].

- What is your impression of what is happening regarding overdose deaths in the state of Rhode Island?
  - What is your understanding/sense of how this crisis impacts your congregation or community?
    - Yes➔ What do you think are the causes of this rise in opioid fatality rates in our communities?
- What are your general views on opioid addiction?
- What has been your experience with opioid addiction disorder in your congregation?
- What is your understanding/sense of ways to treat substance use disorders such as opioid addiction?
- Ever learned of fentanyl?

3. Questions on Harm Reduction

- What do you think are the interventions that would address this crisis?
- This year, the RI Legislature passed legislation permitting the implementation of pilot harm reduction centers. As noted before, this is a place where people can use pre-obtained drugs without fear of arrest or fatal overdose and will have access to services like treatment and recovery support. What are your views about harm reduction centers?
- What comes to mind when you hear of authorized clean needle exchanges to prevent the spread of HIV and Hepatitis?
- What is your opinion on fentanyl test strips to prevent unknowing consumption of fentanyl mixed with other substance use drugs on the Black market?
- How do you feel about the administration of the medication naloxone, which reverses the effect of overdose?

[The evidence is clear that the use of medications to treat opiate addiction is the best way medically to reduce overdose deaths and treat the disease. However, some people have strong feelings against medications. And in particular, the Black community is getting hit so hard that the rates of use of these medications are lower among Blacks in Rhode Island.

That is a bad combination with rates of opioid use increasing and opioids becoming more dangerous with fentanyl. And for Blacks not taking these medications, there are going to be more deaths. This is the trend that will likely continue unless we do something about it; that's why I am here talking to you]

SUPPLEMENT 1
Semi-structured interview guide for Black Rhode Island clergy about their attitude toward Opioid Use Disorder and harm reduction.

- What are your views on medications for opioid use disorder?
- Have you heard of suboxone or buprenorphine? *[suboxone→ combination of buprenorphine and naloxone, is used to treat opiate addiction]*
  - Do you know of anyone on this treatment or who has had experience with this treatment [in your congregation]?
- Does methadone ring a bell?
  - Do you know of anyone on this treatment or who has had experience with this treatment [in your congregation]?
- What do you think of methadone clinics?

4. Questions about stigma

- What kinds of people do you believe are more likely to be affected by/experience substance abuse issues such as opioid use disorder?
  - What are the drivers or vulnerabilities for substance abuse (opioid use disorder)?
  - What are the protective factors for substance abuse (opioid use disorder)? etc., as well as their sense of the extent to which there is a stigma within their community and what kind.
- In terms of addressing opioid addiction, how would you feel about somebody taking medication instead of being completely drug-free?
- Do you have congregants that have opioid addiction?
  - When you counsel your congregants with opioid use disorder (or substance use), have they spoken about the stigma they feel from the church, family, or community?
  - How does stigma prevent people from talking about substance use issues?
- There is often community concern and opposition when a treatment facility, recovery house, recovery center, harm reduction center, or other recovery-related service tries to locate in a neighborhood. What is your view regarding how the church can respond in this situation?
- Do you have any personal biases?

5. Questions about Faith-based opioid intervention

- Do you have any thoughts on intervention methods in which the church can play a role?

  Yes➔
  - How can this intervention be made comfortable for persons experiencing addiction?
  - What structures (physical) or features within churches might facilitate such an intervention?
  - Would you be comfortable sharing how your church might be able to facilitate such an intervention?
  - What are some things that can hinder or prevent such an intervention in a church?

  No➔
  - The Imani Breakthrough Recovery Intervention is a faith-based, person-centered, culturally informed harm-reduction recovery program in churches. This church-based intervention to address the opioid crisis and addiction problems has been effective in Connecticut in the Black American and Hispanic communities.

SUPPLEMENT 1
Semi-structured interview guide for Black Rhode Island clergy about their attitude toward Opioid Use Disorder and harm reduction.

    - What does person-centered mean to you in the context of an OUD intervention?
    - What does culturally informed mean to you in the context of an OUD intervention?
    - What are your thoughts about churches adopting this program?
    - What would it take for a church to adopt this program?

6. Demographics

Congregation

- What is the congregation size?
- What is the gender distribution?
- What is the age range and distribution?
- What is the racial and ethnic demographic distribution?
- Are some of your congregants struggling with opioid use disorder?
- Are some of your congregants experiencing an overdose?
- What community is your church serving geographically?
    - Where do your congregants live?
- What social services might you offer through your church (substance use ministry, interpersonal violence, soup kitchen—community or social service projects ministry?
- What is your denomination?

Clergy

- What would you say is your racial or ethnic identity? Black American, African Immigrant, White, Hispanic, Other
- What is your theological training experience? Seminary, Apprenticeship, Masters, Doctoral
- What is your educational level background? High School, Some College, College, Professional, Masters or Higher
- What is your age range? 18-29? 30-39? 40-49? 50-64? 65+
- How do you self-identify in terms of gender?

7. Closing

- Are there questions you think I should ask that I have not asked or anything else you would like to share?
