# Supplementary material for: Attitudes of Black American Christian church leaders toward Opioid Use Disorder, overdoses, and harm reduction: a qualitative study
Source: Front Psychiatry. 2024 Apr 3;15:1359826. doi: 10.3389/fpsyt.2024.1359826 (PMC11021723; doi:10.3389/fpsyt.2024.1359826)
Supplement: Supplementary file 2 [file Table_2.pdf]

SUPPLEMENT 2

Thematic codes and subcodes grouped by categories, illustrating attitude toward Opioid Use Disorder and Harm Reduction, from interviews with Black Rhode Island clergy, October 2021-January 2022

**CODES**	**Subcodes**
AWARENESS OF OPIOID USE DISORDER (OUD)	▪ Chasing next high<br>▪ Awareness of fentanyl (10 church leaders)<br>▪ Why the increase in opioid deaths among Blacks<br>▪ OUD is also a disease<br>▪ History of opioid addiction<br>▪ OUD is not a willful occurrence<br>▪ Addiction transcends the physical realm
OUD STORIES	▪ Congregants OUD stories<br>▪ Difficult to recover from addiction<br>▪ Miraculous healing from OUD<br>▪ Personal addiction stories<br>▪ Methadone clinic recounts
HARM REDUCTION	▪ Understanding of harm reduction<br>▪ Inner Value Conflicts<br>▪ In favor<br>▪ Harm reduction center views<br>▪ Not in favor
WHY SOME BLACK AMERICANS DO NOT TAKE MOUD	▪ Inconvenience of frequent clinic visits<br>▪ Misinformation<br>▪ Mistrust of the system<br>▪ Limited access to primary care<br>▪ Culturally insensitive environment<br>▪ Physiological and pharmacological challenges
RISK FACTORS FOR OUD	▪ Hopelessness and disappointment<br>▪ Low socioeconomic status<br>▪ Poor home environment<br>▪ Poor home environment<br>▪ Poor choices<br>▪ Behavioral health challenges<br>▪ Disparities in Black communities<br>▪ Genetics and environmental<br>▪ Anybody is fair game<br>▪ Male gender<br>▪ Spiritual or evil attack
PROTECTIVE FACTORS FOR OUD	▪ Christian teachings and values<br>▪ Learning from negative examples<br>▪ Having resilience<br>▪ Higher socioeconomic status<br>▪ Strong support system
CHURCH INTERVENTION COMPONENTS	▪ Church-affiliated OUD recovery ministries<br>▪ Education and resources<br>▪ Church and agency collaborations

SUPPLEMENT 2
Thematic codes and subcodes grouped by categories, illustrating attitude toward Opioid Use Disorder and Harm Reduction, from interviews with Black Rhode Island clergy, October 2021-January 2022

	▪ Spiritual, psychological, and inspirational counseling<br>▪ Community outreach

SOURCE Authors' analysis of study data
