# Supplementary material for: Attitudes of Black American Christian church leaders toward Opioid Use Disorder, overdoses, and harm reduction: a qualitative study
Source: Front Psychiatry. 2024 Apr 3;15:1359826. doi: 10.3389/fpsyt.2024.1359826 (PMC11021723; doi:10.3389/fpsyt.2024.1359826)
Supplement: Supplementary file 3 [file Table_3.pdf]

SUPPLEMENT 3

Thematic codes and some subcodes illustrating attitude toward Opioid Use Disorder (OUD) and Harm Reduction, with selected responses and associated Biblical references, from interviews with Black Rhode Island clergy, October 2021-January 2022

**Thematic CODES and (subcodes)**	**Selected responses**	**Biblical references**
AWARENESS OF OUD (Addiction transcends the physical realm)	There was a woman that Jesus Christ met with in the Bible, and the Bible said the woman was bent over. Jesus Christ said to the woman, “Woman, thou art loose.” Then Jesus Christ, you know, addressed the foundational issue. Anybody who saw that woman thought she had a physical problem because she was bent over. The truth is that Jesus handled the spiritual part of it for her. And now, the physical aspect of it has become easier to tackle.	Luke 13: 10-13
OUD STORIES (Miraculous healing from OUD)	When the Bible says that Jesus of Nazareth was anointed with the Holy Spirit and with the power of God to set men free, it's true. So my experience has been marked by some examples like that.	Luke 4: 16-21<br>Acts 10:38
HARM REDUCTION<br>(In favor of)	So these people accept methadone and Suboxone. Those people are like blind Bartimaeus. They are screaming, “Son of David, have mercy on me and us.” So they will take this thing because they know that if their life is no more under the leadership of the addiction, they can do better with their life. So for me, I accept that.	Mark 10:46-52
HARM REDUCTION (Inner Value Conflicts)	They can be renewed spiritually by the word of God, yes. But, still, here I was, as a pastor working with people with substance dependence and substance abuse problems, [I could not] give them the primary solution, even though I know that the medication has its role to play.	Romans 12: 2
HARM REDUCTION (Inner Value Conflicts)	Our body, the Bible tells us, is the temple of the Holy Spirit. So, in other words, God doesn't want us to abuse our bodies by injecting some of these chemicals. The Bible does not say you should not smoke-- we don't see it anywhere. But when we look at the consequences of smoking in the life of	1 Corinthians 6:19

SUPPLEMENT 3
Thematic codes and some subcodes illustrating attitude toward Opioid Use Disorder (OUD) and Harm Reduction, with selected responses and associated Biblical references, from interviews with Black Rhode Island clergy, October 2021-January 2022

	somebody, that's when we understand that you cannot honor God and at the same time honor something contrary to God, you see?	
RISK FACTORS FOR OUD (Anybody is fair game)	The Bible tells us of Naman in the Bible, a powerful soldier who was a leper, so it cuts across the socio-economic structure. But then it is more prevalent in the people on the lower rung of the ladder.	2 Kings 5:1-14
PROTECTIVE FACTORS FOR OUD (Christian teachings and values)	"Be anxious for nothing, but by prayer and supplication with thanksgiving." He said, "Who can add a single hour to their life?" So that's a lot of stuff that we preach, what we preach to help people understand the truth that then sets them free.	Philippians 4: 6-7<br>Luke 12: 22-26
PROTECTIVE FACTORS FOR OUD (Christian teachings and values)	What we teach is that you have a purpose. You have a destiny. And so when you help people change their ideas, as the scripture says, "As a man thinks, so is he."	Proverbs 23:7a
PROTECTIVE FACTORS FOR OUD (Christian teachings and values)	I use Philippians 4: 7 and 8. So before I go to bed, is this good? Is it pure? Is this praiseworthy? No. Forget it. So, I use scriptures to condition myself. But what about other people who cannot do that? They use other things, drugs, so that they relax them.	Philippians 4:7-8
CHURCH INTERVENTION COMPONENTS (Church-affiliated OUD recovery ministries)	The word of the Lord says that He is doing a new thing. And the Imani Breakthrough Recovery project is a new thing.	Isaiah 43:18-19
CHURCH INTERVENTION COMPONENTS (Church-affiliated OUD recovery ministries)	It means what Jesus demonstrated in the Gospel of John, with the woman at the well. Person-centered speaks of that: You're the most important person on the planet at that moment.	John 4:3-42
CHURCH INTERVENTION COMPONENTS (Education and resources)	So if Jesus were alive today, he would seek out individuals dealing with one issue or the other. Those who are whole don't need a physician, but those who are sick do.	Matthew 9:12
CHURCH INTERVENTION COMPONENTS (Education and resources)	And if I'm honest, I go back to the feeding of the five thousand. Jesus looked at the disciples and said, you feed them. And like the disciples, I think the church too often	Matthew 14:13-21

SUPPLEMENT 3
Thematic codes and some subcodes illustrating attitude toward Opioid Use Disorder (OUD) and Harm Reduction, with selected responses and associated Biblical references, from interviews with Black Rhode Island clergy, October 2021-January 2022

	goes, what do we have? We don't have enough to feed these. And Jesus showed in that example that there was enough. We just needed to know how God would use what we have.	
CHURCH INTERVENTION COMPONENTS (Education and resources)	Even the Bible says that my people perish because they don't know. So when we don't have the right information, we can never make a good decision.	Hosea 4: 6a
CHURCH INTERVENTION COMPONENTS (Education and resources)	Well, the first thing I think we have to do is train our leaders and people to be a welcoming congregation. That the whole Jesus line, come unto me, all you who labor, right? We have to have that "all you who labor" type-- everybody who got something they're dealing with, regardless of what it is, so that they can feel welcomed in the church.	Matthew 11:28
CHURCH INTERVENTION COMPONENTS (Education and resources)	The church has to get some people-- and train them-- who can go outside and do it. And then we can educate the church, and the members, on what is going on in the world today, that drugs and other things have destroyed most people. Jesus says we are the light of the world.	Matthew 5:14-16

SOURCE Authors' analysis of study data
